# Rapid Assessment of Honey Physicochemical Properties by Mid-Infrared Spectroscopy and Chemometric Modeling

**DOI:** 10.1021/acsomega.5c13091

**Published:** 2026-07-22

**Authors:** Roberta R. C. Cangussu, Amanda B. S. Lima, Sibelli P. B. Ferrão, Leandro S. Santos

**Affiliations:** Departamento de Tecnologia Rural e Animal, Universidade Estadual do Sudoeste da Bahia, Itapetinga 45700-000, Bahia, Brasil

## Abstract

This study aims to develop multivariate calibration models for the physicochemical parameters of Brazilian honey samples using mid-infrared (MIR) spectroscopy. A total of 97 samples of honey from *Apis mellifera*bees from different botanical and geographical origins in Brazil were subjected to physicochemical analyses of pH, moisture, total soluble solids, free acidity, lactonic acidity, total acidity, proline, diastatic activity, hydroxymethylfurfural, and MIR. Principal component analysis (PCA) was performed as an exploratory technique to assess the dispersion of the samples across physicochemical parameters. To predict physicochemical parameters of honey from MIR spectral data, spectral interval selection via partial least-squares regression (iPLS) was used. The PCA was performed based on physicochemical parameters, with the samples dispersed according to the quality standards set by Brazilian legislation. MIR spectroscopy, combined with iPLS analysis, allowed the construction of models with high predictive capacity for all parameters evaluated in honey samples. The models presented statistical validation parameters, including correlation coefficients ranging from 0.81 to 0.97, performance ratios for deviation from 1.7 to 4.4, and interval error ratios between 5.7 and 16.3. The results demonstrate the potential of using MIR spectroscopy in conjunction with iPLS for the simultaneous, fast, and nondestructive quantification of physicochemical parameters of honey.

## Graphical abstract

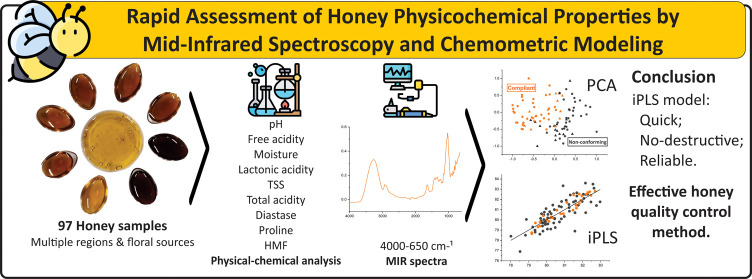

## 1 Introduction

Honey is a natural, nutritious bee product that consumers highly appreciate. It has a high market value, with global production exceeding 1.8 million tons in 2022.^1^ Brazil stands out as the ninth-largest producer in the world and is recognized for supplying high-quality honey.^2,3^

The physical and chemical parameters of honey are set to ensure a safe, high-quality product. Quality honey must be pure, without other ingredients; preserved, showing no fermentation; fresh, with no signs of heating or prolonged storage; and matured in the hives, as indicated by cell capping. Parameters like moisture, pH, free acidity, and total acidity relate to preservation. Diastase (invertase) activity and hydroxymethylfurfural indicate freshness. Proline, moisture, and sucrose content indicate whether the honey was collected ripe or green, meaning it was matured in the hive.^4^

Reference methods are fundamental for assessing honey quality, with physicochemical, chromatographic, and UV–vis spectroscopy analyses playing a central role in quality control. However, these methodologies are generally time-consuming, costly, and complex, with limitations on the number of samples that can be analyzed daily. Given this, there is growing interest in developing faster, more accurate analytical methods to reduce analysis time, expand evaluation capacity, and improve the efficiency of laboratory routines.^5,6^

Infrared spectroscopic techniques have been used in food quality analysis due to their advantages over reference methods. They can obtain spectral information quickly and do not require labor-intensive sample preparation.^7,8^ Midinfrared (MIR) spectroscopy is a technique used to analyze different types of food. Spectral information is obtained in the 4000–400 cm^–1^ region due to changes in molecular rotational and vibrational modes. The bands observed in the spectral data are characteristic of the functional groups in the sample’s chemical composition. Thus, MIR spectroscopy, combined with chemometric tools, is used to extract spectral information, separate and classify samples, and quantify constituents.^9,10^

Infrared analysis has been combined with partial least-squares (PLS) regression to assess honey quality and predict its constituent composition. Studies are being conducted using multivariate calibration algorithms, including full-spectrum models and algorithms for selecting intervals or individual variables, to achieve the best results in constructing multivariate calibration models for food.^11,12^

MIR spectroscopy combined with chemometrics has been used to classify and authenticate honey samples based on their botanical origin^13–15^ and geographical origin,^14,16^ as well as to detect possible adulterants.^8,10,17,18^ In addition, the MIR technique has been used to predict the presence of various compounds in honey. The physicochemical properties of pH, electrical conductivity, free acidity, HMF, fructose, glucose, and moisture were successfully predicted for Romanian honeys.^15^ Anjos et al.^9^ obtained good models for quantifying glucose, fructose, turanose, and melezitose sugars in honey samples from Portugal using MIR spectroscopy. Tahir et al.^19^ predicted phenolic compounds, flavonoids, and antioxidant activity in Sudanese honeys from different botanical sources, while Tahir et al.^20^ quantified the volatile compounds in honey from Sudan.

Although several studies have applied MIR/FTIR spectroscopy for the characterization of honey and the prediction of its constituents, the present study advances the field by employing a structured multivariate strategy based on Interval Partial Least Squares (iPLS) regression, enabling the identification of chemically informative spectral intervals compared to full-spectrum models. Unlike conventional approaches, the SPXY (Sample set Partitioning based on joint X–Y distances) algorithm was adopted to partition the calibration and external validation sets, ensuring simultaneous representativeness of spectral variability (X matrix) and the concentration ranges of physicochemical parameters (Y matrix), which tends to increase predictive robustness and reduce bias associated with purely random partitioning. Furthermore, it stands out for simultaneously modeling multiple physicochemical parameters indicative of quality, using a data set with broad compositional variability arising from the botanical and geographical diversity of Brazilian honey. This integrated approach reinforces the potential of MIR spectroscopy as a rapid, applicable analytical tool for industrial routines that require reliable decision-making. Due to the diversity of honey types produced and, consequently, the variability in their composition, the objective of this study was to develop multivariate calibration models, based on MIR spectral data and chemometrics, for predicting physicochemical parameters in honey samples from different botanical and geographical origins in Brazil.

## 2 Results and Discussion

### 2.1 Physicochemical Analyses

The results of the physical and chemical parameters of the honey samples are presented in Table 1. In relation to the required standards, 51% of the samples presented at least one parameter that did not meet the legal limits for free acidity, moisture, HMF, diastase, and proline.^21–23^ The honey samples were obtained from various botanical and geographical sources, resulting in a diverse composition and high variability in values for each analyzed parameter. Table S1 presents the complete results of the physicochemical analyses of the 97 individual honey samples.

**Table 1:** Results of Physicochemical Analyses of Honey Samples from Different Botanical and Geographical Origins^a^ ^,^ ^b^ ^,^ ^c^ ^,^ ^d^

parameters	average	minimum—maximum	legislation	nonstandard samples (n)
TSS (° Brix)	80.76	76.90–83.60	-^a^	-
moisture (%)	17.50	14.60–21.30	Max. 20^a^	1
pH	4.21	3.38–5.42	-^a^	-
free acidity (meq/kg)	34.04	10.34–72.73	Max. 50^a^	15
lactonic acidity (meq/kg)	7.72	0.00–21.86	-^a^	-
total acidity (meq/kg)	41.76	16.29–84.75	-^a^	-
HMF (mg/kg)	69.45	0.70–339.12	Max. 60^a^	36
diastase (Schade)	14.66	2.37–49.92	Min. 8^a^	28
proline (mg/kg)	476.96	126.65–876.46	Min. 180^a^	3

a TSS: Total soluble solids; HMF: hydroxymethylfurfural.

b Brasil;^(^ ^21^ ^)^.

c International honey comission;^(^ ^23^ ^)^

d No legally established reference values.

In the analyzed honey samples, the TSS content ranged from 76.90° to 83.60° Brix. A high SST content indicates a high concentration of sugars and, consequently, a low moisture content; this parameter is inversely correlated with water content.^24,25^ One of the samples had moisture content above the legal limit (20%) set by Brazilian legislation.^21,22^ Honey moisture is an important quality criterion, as it can indicate incomplete maturation in the combs. Additionally, high water content can promote the growth of molds and yeasts, thereby favoring fermentation and compromising product preservation.^26,27^

Determination of pH is not a legally required parameter,^21,22^ although it is important for assessing honey quality. Acidic pH is desirable because it contributes to product preservation by hindering the growth of microorganisms, as most of them have an optimal pH between 6.5 and 7.5.^25,28^ In this study, the honey samples had acidic pH values, ranging from 3.38 to 5.42.

Regarding free acidity, Brazilian legislation and the Codex Alimentarius establish a maximum value of 50 mequiv per kilogram of honey. A total of 15 samples exceeded this limit, suggesting poor honey preservation and possible fermentation.^21–23^ Free acidity in honey is associated with the presence of organic acids, mainly gluconic acid. There are no established limits for lactonic and total acidity, with lactonic acidity being considered the acidity reserve when honey becomes alkaline, and total acidity being the sum of free and lactonic acidity.^29,30^

HMF content and diastase activity are two parameters used to assess honey freshness. Honey stored for a long time, improperly stored, or heated has a high HMF content and low diastase activity.^31^ According to Brazilian legal standards, 37% of the samples had HMF content above the recommended level, and 29% of the samples had diastase activity below the legal limit, indicating a deficiency in the freshness of the samples.^21^

Although it is not a quality parameter established in Brazilian law, proline content is an important attribute considered in different countries. Proline levels below 180 mg/kg may indicate that the honey has not fully matured and may also suggest possible adulteration.^23^ In the present study, three samples had proline content below 180 mg/kg.

Among the parameters analyzed, HMF (37%), diastase activity (29%), and free acidity (15%) stand out as the parameters with the highest number of samples outside the legal limits. The presence of honeys with parameters outside the recommended values is undesirable from both legal and quality-control perspectives. However, to generate mathematical models with broad practical applicability, it is desirable to have data sets with high variability across samples, including honeys with different geographical and botanical origins, quality conditions, and storage conditions, so that these models are robust and satisfactorily cover diverse honey samples.

### 2.2 Principal Component Analysis of Physicochemical Parameters

PCA was performed as an exploratory analysis of the data, reducing the number of variables used to evaluate honey quality. PCA enabled visualization of the distribution of the 97 samples across physicochemical parameters (Figure 1). Two principal components were used to describe the data’s variability, with the first principal component (PC1) accounting for 49.05% of the variance. In contrast, the second principal component (PC2) accounted for 19.45% of the variance in the data. PC1 showed an inverse correlation with honey quality, as the variables free acidity, total acidity, lactonic acidity, moisture, and HMF are positively correlated with PC1, whereas pH and TSS are negatively correlated with it. Regarding PC2, the variables proline and diastase showed a high positive correlation. Thus, it is expected and has been observed that higher-quality honey samples, with parameters within legal limits, show dispersion and concentration on the upper-left side of the dispersion graph. Samples that showed at least one physicochemical property outside legal limits tended to concentrate on the right side, exhibiting a positive correlation with PC1.

**Figure 1:**
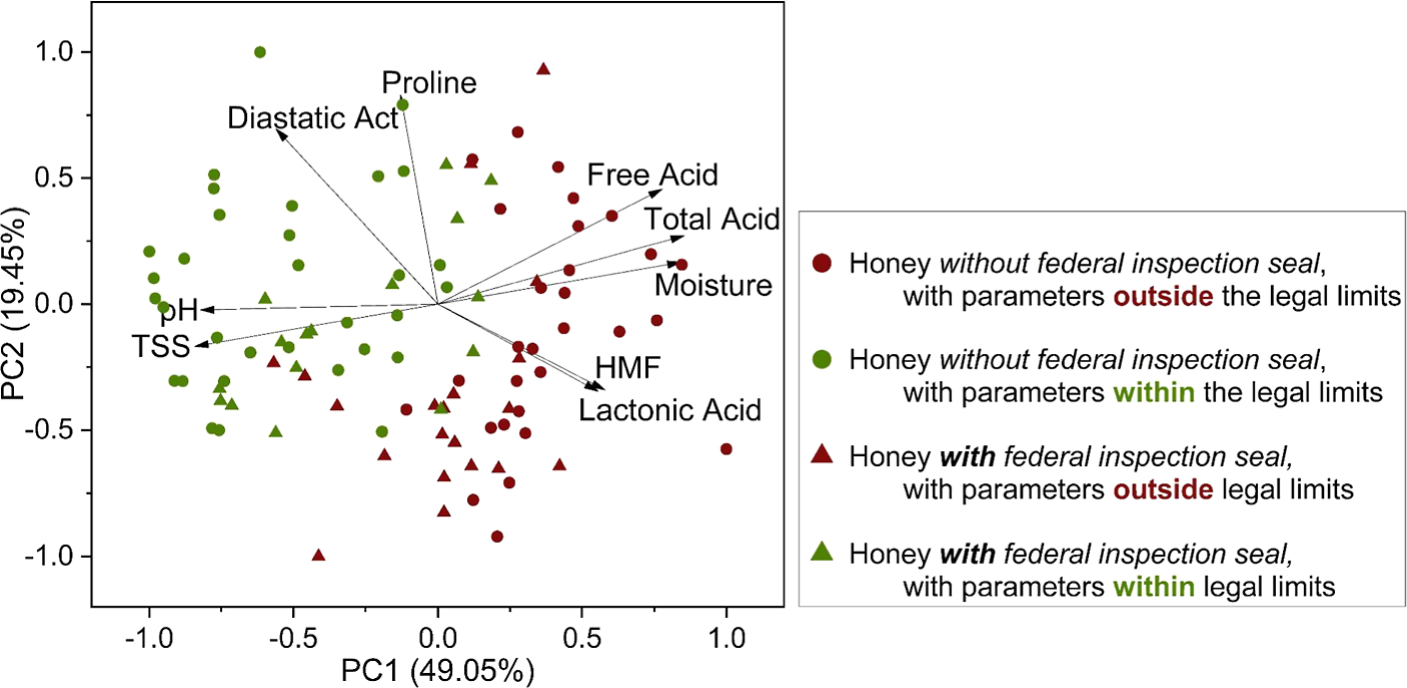
Dispersion of honey samples within and outside the limits stipulated by legislation, based on data from physicochemical analyses and principal component analysis.

Through PCA, it is also possible to suggest that the possession of a federal inspection seal, despite being a basic legal requirement for the commercialization of the product, does not guarantee the quality of the honeys sold. This fact is demonstrated by the dispersion of the samples, where inspected honeys resemble those without an inspection seal, and mainly by the number of inspected samples that showed parameters outside the legal standards.

These observations demonstrate the urgency of improving the inspection system for beekeeping establishments and products in accordance with the stipulated terms. And it also allows presenting PCA as a statistical screening tool for honeys, based on physicochemical data, for quality and compliance with legal standards.

Despite this, performing this screening requires conducting all the benchtop analyses presented, which makes the process expensive and time-consuming. An alternative to circumvent this scenario is the use of infrared spectroscopy techniques, which, through multivariate statistics, allow the simultaneous prediction of several chemical parameters in a fast, efficient, and reliable way. This tool has been applied to various food products, including bee products such as honey, using other spectroscopic techniques, or to detect adulteration, propolis, or other food products.^46–52^

### 2.3 Midinfrared Spectroscopy

The mid-infrared region (4000–650 cm^–1^) provides information on the molecular vibrations in the samples, enabling assessment of the chemical composition of honey.^15^ The spectra of the honey samples exhibited characteristic bands with varying absorption intensities, enabling the prediction of compounds and properties in honey using multivariate tools. The spectra obtained from the MIR absorbances of the honey samples are shown in Figure 2. Thirteen spectral bands were identified: 3290, 2937, 2892, 1648, 1421, 1342, 1261, 1148, 1024, 920, 865, 818, and 777 cm^–1^.

**Figure 2:**
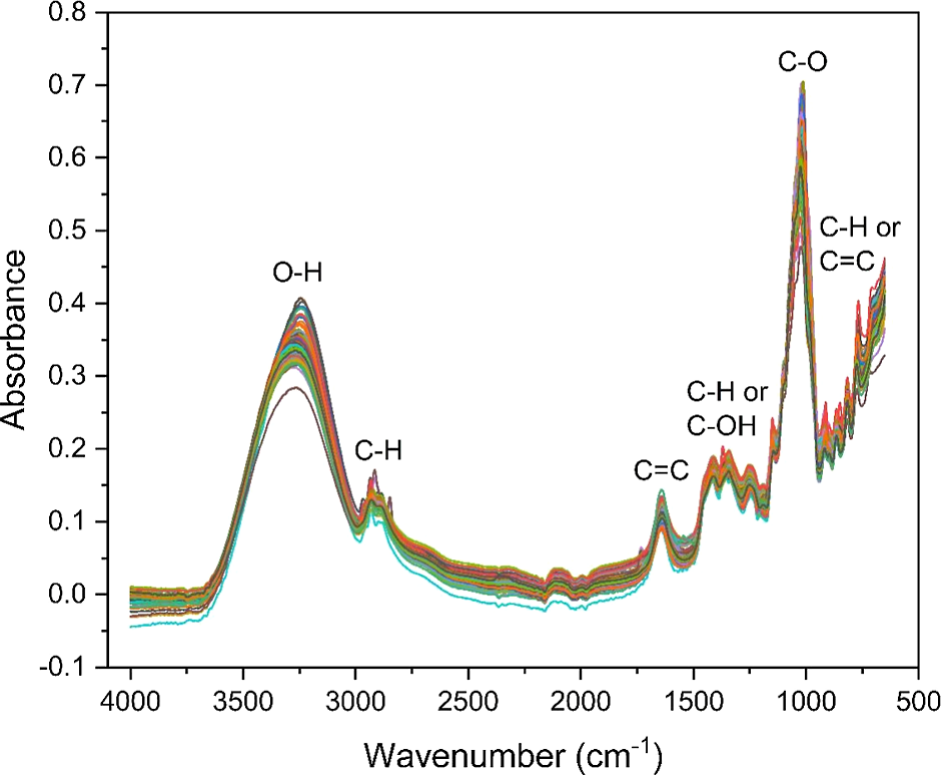
Midinfrared spectra of honey (4000–650 cm^–1^).

The band around 3300 cm^–1^ is associated with the stretching vibration of the O–H group present in water and the stretching vibration of the O–H group present in honey carbohydrates. The two bands around 2900 cm^–1^ were associated with the C–H stretching vibrations of carbohydrates and the (NH_3_) stretching vibrations of free amino acids (present in low concentrations in honey). The band at 1648 cm^–1^ is associated with the presence of water, due to the deformation vibration of the O–H group. The region between 1474 and 1199 cm^–1^ is attributed to the vibrations of the C–C–H, C–O–H, and O–C–H groups, while the region between 1153 and 950 cm^–1^ is associated with the stretching vibrations of the C–O (C–OH) and C–C groups present in the carbohydrate structure. The band at 1421 cm^–1^ may be due to a combination of vibrations of the O–H (C–OH) group and C–H deformation in alkenes. The band around 1342 cm^–1^ is associated with O–H (C–OH) vibrations. The region between 950 and 700 cm^–1^ is related to the anomeric region of carbohydrates and C–H and C=C deformation vibrations of carbohydrates.^9,10,18,32–35^

### 2.4 *i*PLS Analysis

*i*PLS analysis was performed to evaluate the predictive capacity of MIR spectral data for determining the physical and chemical parameters of honey samples. The calibration data set was intentionally designed to include substantial botanical, geographical, temporal, and commercial variability in order to improve the robustness and practical applicability of the multivariate models. Although the number of samples was moderate, the broad variability represented in the data set increases the chemical representativeness of the calibration space and better reflects real-world honey heterogeneity. A total of nine parameters were calibrated, and the models were adjusted accordingly. For each parameter evaluated, the spectra were partitioned into intervals containing 20–55 variables. The calibrated models selected between 5 and 13 significant intervals for adjustments. The greatest reduction was observed when iPLS was used to predict HMF content, with the number of variables reduced from 14,000 to 160, and the models with the best adjustment parameters were selected. The use of variable selection methods enables calibration of models using only the most relevant variables, thereby reducing the introduction of spectral noise, improving statistical analysis time, and enhancing model adjustment parameters. Figures S1–S9 show the intervals used to construct the models for each parameter. Table 2 presents the statistical parameters of the calibration and validation data sets for the best model of each physicochemical parameter evaluated.

**Table 2:** Adjustments of the iPLS Models for the Calibration and Validation Dataset of the Physicochemical Parameters of Honey Samples from Different Botanical and Geographical Origins Using Mid-infrared Spectroscopy Data^a^

para-meters	varia-ble by interval	intervals selected	LV	calibration					validation				
				*R*	RMSE	RPD	RER	Bias	R	RMSE	RPD	RER	Bias
pH	55	5	9	0.83	0.28	1.80	7.22	0.00	0.93	0.16	2.83	10.49	0.00
free acidity	25	10	13	0.65	11.00	1.33	5.67	0.00	0.85	4.44	2.02	7.51	–1.43
moisture	50	9	12	0.88	0.67	2.16	10.03	0.00	0.94	0.45	2.91	9.46	–0.03
lactonic acidity	50	10	10	0.79	3.09	1.65	7.08	0.00	0.97	1.28	4.39	16.33	0.18
TSS	25	8	13	0.79	0.85	1.65	7.89	0.00	0.95	0.39	3.34	10.76	0.06
total acidity	30	13	5	0.68	11.98	1.38	5.72	0.00	0.81	7.01	1.75	5.72	–0.66
diastase	25	12	13	0.72	6.52	1.46	7.29	0.00	0.96	2.41	3.80	10.93	–0.55
proline	25	7	13	0.66	146.46	1.33	5.12	0.00	0.85	86.03	1.93	7.81	–5.75
HMF	20	8	13	0.56	73.89	1.21	4.57	0.00	0.85	17.94	1.97	6.15	–2.04

a TSS: Total soluble solids; HMF: Hydroxymethylfurfural; LV: number of factors; *n*: number of samples; *R*: correlation coefficient; RMSE: root-mean-square error; RPD: performance-to-deviation ratio; RER: range error ratio.

To evaluate the model’s efficiency, the statistical parameters R, RMSE, RPD, and RER were used. A good prediction model should have an *R* value close to 1 and a low RMSE. The RPD parameter >2.0 indicates that the model is excellent; 1.4 < RPD <2.0 indicates that the model is reasonable; and RPD <1.4 indicates an unreliable model. The RER >10 adjustment parameter indicates good practical utility, 3 < RER <10 indicates moderate utility, and RER <3 values indicate low predictive ability.^35–37^

The calibration models presented R values ranging from 0.56 to 0.88. Some of the models have values below 0.7, which is the minimum acceptable for a well-adjusted model. However, this profile improves when the models are validated with a set of external samples, obtaining validation *R* values ranging from 0.81 to 0.97. These data indicate good generalization and predictive capacity for the models. According to Ferreira,^38^ this is the ideal behavior exhibited by a well-adjusted model, in which the validation parameters yield better values than the calibration parameters, thereby avoiding underfitting or overfitting and demonstrating the high practical application capacity of the models. Thus, to analyze the efficiency and adjustments of the calibrated models, the adjustment data for the validation sets will be considered.

The external validation performed in this study employed independent samples selected through the SPXY algorithm, ensuring representative distribution of spectral and analytical variability between calibration and validation sets. Thus, approximately 30% of the samples were excluded from model development and used exclusively for external prediction. Although all honey samples originated from Brazil, the data set encompassed substantial variability related to botanical origin, geographic region, climatic conditions, harvest periods, and commercial source. Considering the continental dimensions and biodiversity of Brazil, this variability contributes to broad chemical representativeness of the calibration models. Nevertheless, as with most spectroscopy-based calibration studies, extrapolation to samples from entirely different global production contexts should be performed cautiously.

For comparison with other machine learning approaches, the validation set data for calibration using the standard PLS algorithm are presented in the Supporting Information (Table S2). These data allow assessment of the contribution of interval variable selection through iPLS to model performance. The iPLS approach improved the fitting and predictive ability of all models, particularly for free acidity, proline, and HMF, whose full-spectrum PLS models presented correlation coefficients of 0.01, 0.29, and 0.01, respectively. These results highlight the importance of spectral variable selection to reduce noninformative regions and improve calibration performance in complex honey matrices.

According to the adjustment parameters for *i*PLS, the models developed to predict pH, lactone acidity, SST, and diastatic activity demonstrated excellent predictive ability, particularly the model for lactone acidity, with R values of 0.97, RPD of 4.39, and RER of 16.33. The models calibrated for the proline and HMF parameters presented greater complexity and difficulty in efficient prediction. This was expected due to the low concentrations of these analytes in the honey samples, which were further aggravated by the wide range of values found, making the construction of the models challenging but with high robustness and practical applicability.

The honey samples used in this study were collected in different geographical regions, with different botanical origins and production times. Consequently, these samples exhibit heterogeneous characteristics, as reflected in the wide range of physical and chemical parameters. Therefore, constructing multivariate calibration models for honey quality parameters using MIR is challenging due to the diversity of the samples; however, it yields models with a wide range of applications and high reliability, not restricted to a single type of honey.

Thus, the suitability and practical implications of the models can be critically evaluated for these more complex calibration parameters, considering not just one figure of merit but the entire set. In this sense, although the models for free acidity, total acidity, proline, and HMF presented low correlation coefficients for calibration (0.56 to 0.68) and good values for validation (0.81 to 0.85), they need to be evaluated in conjunction with RMSE, RPD, and RER data, which indicate models with moderate practical utility and reasonable predictive capacity. Therefore, these models should be used with screening techniques to verify the suitability of these parameters for meeting quality standards, and should not be used as the primary tool for determining analytical concentration. For the other models not mentioned, they can be used as screening and predictive tools for analytical values.

Figure 3 illustrates the correlation between the reference and predicted values for the calibration and validation data sets of the developed models. These data illustrate what the correlation coefficient numerically indicates and show the best fit for the validation data sets. Additionally, the figure displays the intercept and slope data for the models, indicating the linearity of the fits and providing further evidence of the model’s quality. The data show excellent fits within the calibration range of the models, with intercepts close to 0 and slopes close to 1.

**Figure 3:**
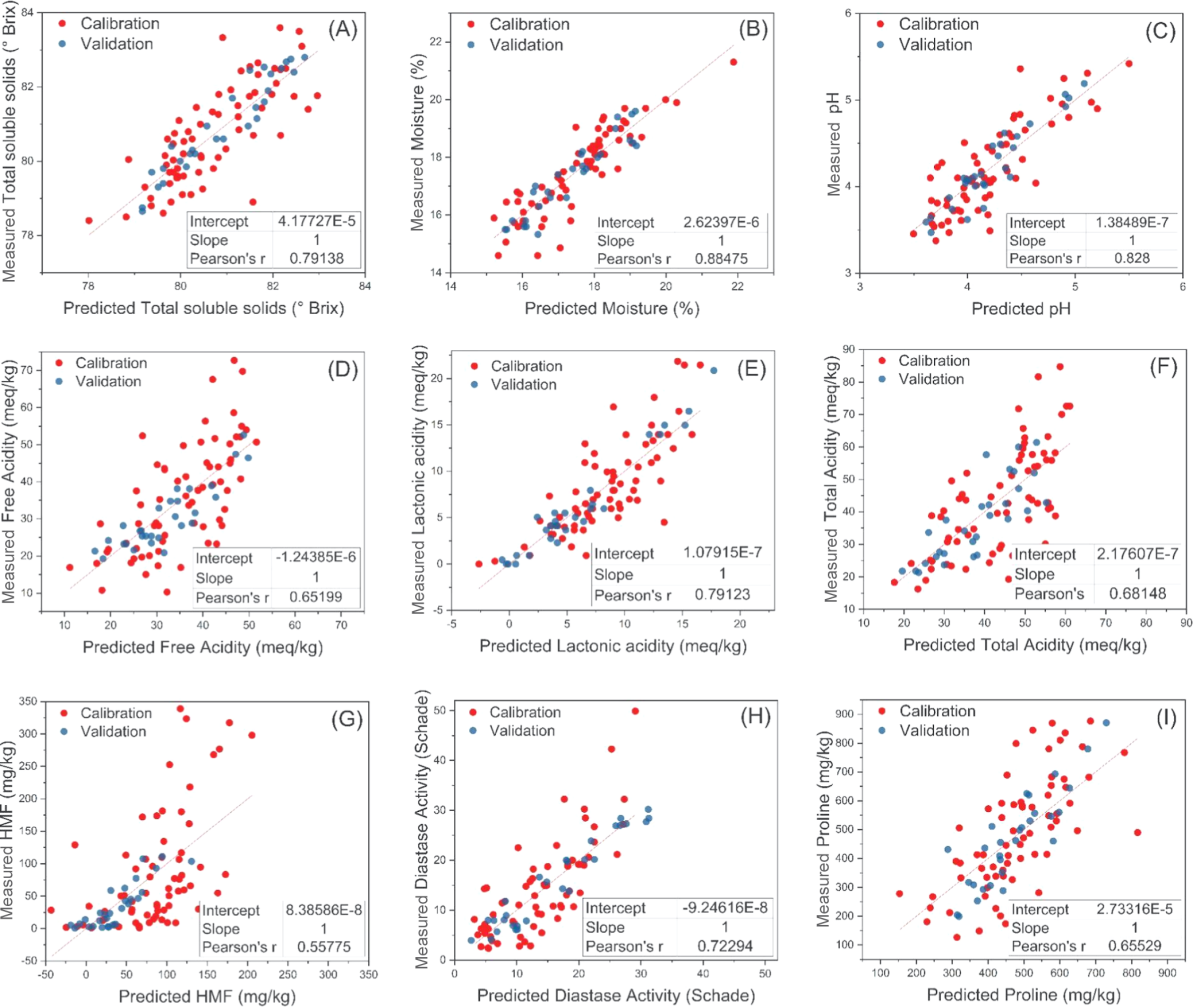
Correlation between reference values and values predicted by *i*PLS for calibration and validation data of the physicochemical parameters of honey samples from different botanical and geographical origins using mid-infrared spectroscopy data for: A - total soluble solids; B - Moisture; C - pH; D - free acidity; E - lactonic acidity; F - total acidity; G - hydroxymethylfurfural; H - diastase activity and; I - proline.

The iPLS algorithms effectively selected optimal subintervals from all spectra. Using MIR spectroscopy, it is possible to perform rapid, simultaneous analyses of nine quality parameters of Brazilian honey from different geographical and botanical origins, and varying degrees of freshness. Four of these generated models can be used as screening tools to indicate whether the quality parameters (total acidity, free acidity, proline, and HMF) are met, due to limitations in the detection and quantification techniques of mid-infrared spectroscopy. Meanwhile, five of the generated models (pH, moisture, lactonic acidity, TSS, and diastatic activity) can serve as predictive tools for the simultaneous assessment of analytical quality levels in honey, reducing the time required and the complexity of honey quality control by regulatory bodies, industries, and producers.

Additionally, the computational requirements of the proposed MIR-iPLS methodology are primarily concentrated in the model development stage, which is performed offline. Model calibration and interval selection were conducted using the open-source R environment and do not require specialized hardware or proprietary software. The final models were developed from spectra containing on average 300 spectral variables, partitioned into on average 9 intervals for iPLS evaluation. The complete model development procedure required approximately 6 min on a standard desktop computer equipped with processor 12th Gen Intel­(R) Core­(TM) i5-1235U (1.30 GHz), 8GB of RAM, Intel­(R) UHD Graphics (128 MB) graphics card. Once the calibration models are established, routine implementation involves only spectral acquisition, spectral preprocessing, and application of the previously optimized prediction equations. The prediction time for a new sample was approximately 30 s, and no iterative optimization or model retraining is required during routine analysis.

From an operational perspective, the methodology enables the simultaneous estimation of 9 physicochemical parameters from a single MIR spectrum, reducing the number of analytical procedures required compared with conventional laboratory methods. Spectral acquisition required approximately 45 s per sample, with minimal or no sample preparation. Combined with the nondestructive nature of MIR spectroscopy, these characteristics support its applicability in routine quality-control environments. Furthermore, the use of a single spectral measurement to obtain multiple quality parameters may contribute to reductions in analytical workload and overall processing time compared with conventional laboratory procedures, which typically require 9 separate analyses and approximately 2 days per sample.

### 2.5 Model Transferability and Methodological Limitations

The robustness of the proposed models is supported by the compositional diversity of the calibration data set. The study included honey samples from distinct geographical regions, different harvest periods, and varied botanical origins. Such heterogeneity increases spectral and physicochemical variability, allowing models to capture a broader range of compositional patterns and reducing the risk of overfitting to a single production context.

Geographic origin influences mineral composition, climatic conditions, and floral availability, while seasonal variations affect moisture equilibrium and minor constituents. Botanical source, in turn, directly impacts carbohydrate profile, organic acids, and secondary metabolites, all of which contribute to mid-infrared spectral variability. By incorporating this natural diversity during calibration, the models were constructed to reflect realistic variability found in commercial honey production. Nevertheless, despite this intrinsic robustness, direct transferability to honeys from substantially different countries, climatic zones, or production systems should be approached with caution. Expanding compositional domains beyond those represented in the current data set may introduce spectral patterns not fully captured by the existing calibration space.

Instrumental factors also require consideration. FTIR-ATR measurements are influenced by crystal characteristics, applied pressure, spectral resolution, and instrument-specific response. Differences between spectrometers or ATR accessories may introduce systematic spectral shifts, potentially affecting predictive performance if models are transferred without recalibration or standardization procedures. Additionally, ATR-based measurements are sensitive to sample homogeneity, temperature control, and penetration depth, which may introduce variability if operational conditions are not rigorously standardized.

This study focused specifically on evaluating MIR spectroscopy associated with iPLS for prediction of honey quality parameters and was not designed as a comparative assessment between different spectroscopic techniques or portable analytical devices. Comparative studies involving NIR spectroscopy, hand-held instruments, or other rapid analytical platforms may provide complementary information regarding operational applicability and could be explored in future investigations.

For these reasons, the proposed models should be considered active calibration models. Periodic updating with new representative samples, monitoring of prediction residuals, and recalibration when necessary are recommended to maintain long-term reliability. Incorporating new seasonal batches or samples from additional geographic regions is expected to further strengthen model generalizability and predictive stability over time.

## 3 Conclusion

Midinfrared spectroscopy is a rapid, versatile technique that enables the evaluation of multiple honey parameters in a single test. The results demonstrate the feasibility of using MIR spectroscopy in combination with iPLS for the simultaneous quantification of honey’s physical and chemical parameters, indicating the potential of the method as an alternative or complementary analytical tool for routine quality control.

The robustness of the models was supported by the compositional diversity of the data set, which included honeys from different geographical regions, harvest periods, and botanical origins. This variability allowed the calibration space to encompass realistic compositional differences encountered in commercial production, enhancing model stability.

While certain parameters demonstrated predictive performance suitable for quantitative applications, others are more appropriately interpreted as screening tools. These distinctions are essential for defining the practical scope of implementation in quality monitoring laboratories.

Models were built with high predictive capacity for pH, lactonic acidity, moisture, SST, and diastatic activity, and, as a screening tool, for free acidity, total acidity, proline, and HMF in honey samples from different botanical and geographical origins in Brazil.

This study addresses a fundamental challenge in honey quality control by applying rapid, nondestructive analytical tools that simultaneously predict multiple chemical parameters required by legislation or associated with honey quality and freshness. This new approach requires minimal sample preparation, eliminates the need to handle or consume chemical reagents, and uses a single piece of equipment.

The proposed models are already suitable for implementation in routine procedures, such as those involved in receiving raw materials, where quick decisions are necessary. But should be regarded as dynamic calibration systems, capable of continuous improvement as additional data becomes available.

## 4 Methods

### 4.1 Acquisition of Honey Samples

Between 2019 and 2022, 97 samples of honey from *Apis mellifera* bees of different botanical origins, produced in Brazil, were acquired. The samples were produced in the states of Minas Gerais (*n* = 59), Bahia (*n* = 35), Espírito Santo (*n* = 1), Paraíba (*n* = 1), and Maranhão (*n* = 1). The samples were purchased directly from local producers (*n* = 37) and in retail stores (*n* = 60). Among the samples purchased, 37 had the Brazilian Federal Inspection Seal (SIF). Regarding botanical origin, the honeys were declared as multifloral and/or wild honey (*n* = 36), aroeira honey (*n* = 42), eucalyptus honey (*n* = 5), açaí honey (*n* = 1), coffee honey (*n* = 1), assa peixe honey (*n* = 1), cat’s claw honey (*n* = 1), and honey with no identification of botanical origin (*n* = 10). The honey samples analyzed were less than 24 months old at the time of production.

### 4.2 Determination of pH and Acidity

To determine the pH, 10 g of honey were dissolved in 75 mL of distilled water, and the pH was measured directly with a digital pH meter (Quimis, Diadema, SP, Brazil). To analyze the free acidity, the honey solution was titrated with a 0.05 N sodium hydroxide solution (Dinâmica Química Contemporânea Ltd.a., Indaiatuba, SP, Brazil) to a pH of 8.5. Then, 10 mL of 0.05 N sodium hydroxide solution was added, and the mixture was with 0.05 N HCl solution (Anidrol, Diadema, SP, Brazil) to a pH of 8.3 for analysis of lactonic acidity. Total acidity was obtained from the sum of free and lactonic acidity. The acidity results were expressed in meq/kg.^39^

### 4.3 Determination of Moisture and Total Soluble Solids

The determination of moisture and total soluble solids (TSS) was performed by the refractometry method, using an Abbé refractometer (Quimis, Diadema, Brazil) coupled to a thermostatic bath (Tecnal, Te-184, Piracicaba, Brazil). For the analysis, approximately two drops of honey were placed on the refractometer prism, and the refractive index value at 20 °C was converted to moisture percentage using the Chataway table. To determine TSS, a direct reading was taken on the equipment, and the result was expressed in °Brix.^23^

### 4.4 Proline Determination

Proline determination (Êxodo Científica, SP, Brazil) was performed using the official AOAC 979.20 (2006).^40^ An aqueous honey solution (50 mg/mL) was prepared, and 0.5 mL of the honey solution, 0.5 mL of 85% formic acid, and 1.0 mL of ninhydrin solution (Química Moderna, Barueri, SP, Brazil) were added to three test tubes. The tubes were placed in a boiling water bath for 15 min and then cooled in a water bath at 22 °C. Five milliliters of an aqueous isopropanol solution (1:1) (Química Moderna, Barueri, SP, Brazil) was added, and the absorbance was measured at 520 nm on a spectrophotometer (UV-1800, Shimadzu, Germany) 35 min after removing the tubes from the boiling bath. The results were determined using a calibration curve of a standard proline solution (5 to 50 μg proline.mL^–1^) and expressed in milligrams of proline/kg of sample^–1^.

### 4.5 Determination of Hydroxymethylfurfural (HMF)

To determine the HMF content, 5 g of honey was dissolved in 25 mL of distilled water, and the solution was transferred to a 50 mL volumetric flask. 0.5 mL of Carrez I solution and 0.5 mL of Carrez II solution were added, and the volume was made up to 1 mL with distilled water. The solution was filtered with qualitative filter paper (Unifil, 110 mm, Brazil), and the first 10 mL of the filtrate was discarded. 5.0 mL was pipetted into two test tubes, 5.0 mL of water (sample solution) was added to the first, and 5.0 mL of 0.2% sodium bisulfite solution (Synth, Diamdema, SP) (reference solution) was added to the second. The tubes were shaken well, and the absorbance of the sample solution relative to the reference solution was determined at 284 and 336 nm using a spectrophotometer (UV-1800, Shimadzu, Japan). The HMF content was calculated according to eq 1.^23^

1 $$HMF\left(\frac{mg}{kg}\right)=\frac{\left(A_{284}-A_{336}\right)x149.7x5}{P}$$

where, *A*_284_ = absorbance reading at 284 nm; *A*_336_ = absorbance reading at 336 nm; *P* = sample mass in grams.

### 4.6 Determination of Diastatic Activity

To determine diastatic activity, 10.0 g of honey was dissolved in 15 mL of water, and 5 mL of acetate buffer (pH 5.3) was added. The solution was transferred to a 50 mL volumetric flask containing 3 mL of 0.5 M sodium chloride solution, and the volume was adjusted to the mark with distilled water. A beaker containing 10 mL of the honey solution and another containing 10 mL of the starch solution were placed in a water bath at 40 °C. After 15 min, 5 mL of the starch solution was pipetted into the honey solution, and the stopwatch was started. At regular intervals, 0.5 mL aliquots were removed and added to 5 mL of diluted iodine solution (0.00035 M), and the absorbance was read at 660 nm on a spectrophotometer (UV-1800, Shimadzu, Germany). A standard curve of absorbance versus time (minutes) was constructed, containing 3 to 4 absorbance values ranging from 0.465 to 0.155. Subsequently, the reaction time to reach an absorbance of 0.235 was calculated from the regression equation. 300 was divided by the time obtained, and the result was expressed in Schade units per gram of honey.^23^

### 4.7 Mid-Infrared Spectroscopy—MIR

Spectroscopic analysis was performed on honey samples using Fourier Transform Infrared Spectroscopy with Attenuated Total Reflection (FTIR-ATR) (Cary 630 FTIR, Agilent Technologies Inc., CA, USA). The spectra were obtained over 4000–650 cm^–1^ with 64 scans and a spectral resolution of 4 cm^–1^. The spectra were acquired using Agilent MicroLab PC and Resolution Pro software. For the analysis, the laboratory room temperature was maintained at approximately 25 °C, and around 0.3 mL of the honey sample was placed on the surface of the diamond crystal, yielding the spectra in absorbance mode. Before reading the samples, a blank analysis was performed by scanning the diamond surface without a sample.

### 4.8 Statistical Analysis

#### 4.8.1 Principal Component Analysis (PCA)

PCA was performed as an exploratory analysis of the data to assess the dispersion of the honey samples according to physicochemical quality parameters and to identify possible correlations among the variables. Data on the physicochemical parameters of the honey samples were used to perform PCA. The analysis was performed using a correlation matrix to obtain eigenvalues and eigenvectors, from which principal component scores for each sample were determined. The Statistical Analysis System (SAS) On Demand for Academics statistical program was used to perform the PCA, selecting the PROC PRINCOMP procedure.

#### 4.8.2 iPLS Analysis

The interval partial least-squares (iPLS) regression algorithm was used to build prediction models. The nine physicochemical parameters of honey were used as the dependent variable, and the MIR spectral data were used as the explanatory variable to verify the potential of MIR to predict honey quality parameters as a substitute for reference methods.

Different spectral preprocessing strategies were preliminarily evaluated during model development, including common treatments applied in MIR spectroscopy. However, no significant improvements in calibration or validation performance were observed compared to the original spectra. Therefore, the models presented in this study were developed using untreated spectral data to avoid unnecessary data manipulation and possible introduction of artifacts.

Data analysis was performed using MATLAB R2012b (MathWorks, Natick, MA, USA). For model development, samples were partitioned into calibration (70%) and external validation (30%) subsets using the SPXY (Sample set Partitioning based on joint X–Y distances) algorithm.^41^ SPXY selects samples based on Euclidean distances computed jointly in the predictor space (X matrix, spectral variables) and response space (Y matrix, physicochemical reference parameters), ensuring that both spectral diversity and analytical concentration ranges are represented in the calibration subset.

The algorithm begins by selecting the two most distant samples in the joint X–Y space and iteratively includes samples that maximize multivariate diversity. This approach reduces sampling bias associated with random splitting and enhances the robustness of model calibration. The distribution of calibration and validation samples was subsequently inspected in PCA score space to confirm that both subsets covered the main spectral variance structure without systematic clustering.

To remove outliers, the steps proposed by Botelho et al.^42^ and Valderrama et al.,^43^ were followed, considering extreme leverage and residuals for both the calibration and validation sets.

The iPLS algorithm is a variable selection method that divides the data sets into equal-sized intervals, analyzes them individually and in combinations, and identifies the optimal interval combination for predicting a response variable. The models were generated in RStudio software using the MDATOOLS package with the Forward algorithm.^44^ Preliminary tests were conducted to determine the optimal number of samples per interval for each response variable. The models showed better fits when divided into intervals of 20–55 samples each.

For selecting of intervals and the number of latent variables, the algorithm considers the smallest number of interactions that yield improvements, as indicated by RMSE and *R* ^2^ values. If there is no significant difference, the model with the fewest estimated components and that explains the response variable well is chosen. After selecting the intervals and number of latent variables, the generated iPLS model was used to predict the response variables in the validation data set.

The statistical parameters correlation coefficient (*R*), root-mean-square error (RMSE), performance-to-deviation ratio (RPD), and range error ratio (RER) were used to evaluate the model’s performance for the calibration and validation data, as described by Lima et al.^45^ and Lima et al.^46^ In addition, slope and intercept data from the adjustments were determined to evaluate the linearity of the models.^42,43^ Final model performance was assessed using the independent external validation set.

## Supplementary Material



## Notes

A supplementary file is available containing the results table for the physicochemical analyses of the 97 honey samples, and figures showing the spectral regions selected by iPLS to predict each physicochemical parameter of the honey samples from MIR spectroscopy data (PDF)
