# Supplementary material for: Rapid Assessment of Honey Physicochemical Properties by Mid-Infrared Spectroscopy and Chemometric Modeling
Source: ACS Omega. 2026 Jul 22;11(30):44682–91. doi: 10.1021/acsomega.5c13091 (PMC13449194; doi:10.1021/acsomega.5c13091)
Supplement: Supplementary file 1 [file ao5c13091_si_001.pdf]

Rapid Assessment of Honey Physicochemical Properties by Mid-Infrared Spectroscopy and Chemometric Modeling

Roberta Ribeiro da Cruz Cangussu[1] - cangussu.roberta@gmail.com

Amanda Beatriz Sales de Lima[1] - amandaengalimentos@hotmail.com

Sibelli Passini Barbosa Ferrão[1] - sferrao@uesb.edu.br

Leandro Soares Santos[1]* - leandrosantos@uesb.edu.br

Author affiliation(s)

[1]Departamento de Tecnologia Rural e Animal, Universidade Estadual do Sudoeste da Bahia, Itapetinga, Bahia, Brasil

***Correspondence author:** Leandro Soares Santos, Departamento de Tecnologia Rural e Animal, Universidade Estadual do Sudoeste da Bahia, Praça Primavera - 40, Bairro Primavera, 45700-000, Itapetinga, Bahia, Brasil. Email: leandrosantos@uesb.edu.br

Supplemental Material

Table S1. Results of the physical-chemical analyses of the 97 honey samples.

Samples	pH	Free Acidity (meq/kg)	Lactonic Acidity (meq/kg)	Total Acidity (meq/kg)	Moisture (%)	TSS (%)	HMF (mg/kg)	Proline (mg/kg)	Diastase (Schade)
1	3.57	72.73	8.94	81.67	18.40	79.70	268.26	560.16	5.36
2	3.47	31.58	10.93	42.51	18.20	80.00	109.87	269.22	9.12
3	3.72	24.88	12.91	37.80	16.50	81.80	111.08	541.94	8.00
4	4.14	34.45	9.93	44.39	17.80	80.40	103.58	460.12	6.17
5	3.67	44.99	10.93	55.91	21.30	76.90	79.78	351.12	6.31
6	3.84	28.71	20.86	49.57	17.20	81.10	113.16	148.83	3.94
7	3.60	39.24	7.95	47.19	18.10	80.10	180.07	212.46	7.80
8	4.06	30.63	8.94	39.57	18.50	79.80	252.77	235.15	5.49
9	3.87	50.73	21.86	72.58	20.00	78.40	298.17	278.33	2.37
10	3.87	22.01	9.93	31.95	18.60	79.60	117.03	173.85	3.65
11	3.61	40.20	7.95	48.14	17.60	80.70	218.22	414.66	3.84
12	4.58	26.80	7.95	34.75	17.30	81.00	54.78	582.88	15.00
13	3.85	49.77	7.95	57.72	19.30	78.90	317.36	530.53	7.96
14	4.10	44.02	11.92	55.94	19.20	79.10	37.27	578.36	7.34
15	4.60	20.10	6.95	27.05	17.60	80.60	27.54	546.48	8.06
16	3.78	51.68	5.96	57.64	19.90	78.50	28.59	682.93	14.51
17	4.90	18.19	6.95	25.14	16.30	81.80	5.39	578.29	14.32
18	4.49	21.29	7.45	28.74	18.30	79.90	3.89	471.55	12.92
19	3.81	32.56	11.48	44.04	18.27	80.03	161.65	448.34	4.74
20	4.28	52.11	17.97	70.08	18.73	79.60	21.03	334.99	10.65
21	4.17	29.77	21.46	51.23	16.77	81.50	92.22	455.44	6.33
22	3.73	37.68	5.99	43.67	17.67	80.60	172.00	338.53	2.44
23	3.99	41.40	21.46	62.86	17.40	80.80	173.79	333.19	2.69
24	4.08	17.68	12.48	30.15	17.60	80.75	14.00	390.33	8.18

25	4.23	38.14	6.49	44.63	14.87	83.33	35.40	780.47	20.01
26	4.05	58.61	13.97	72.59	18.50	79.80	34.65	435.84	11.70
27	4.03	69.78	14.97	84.75	18.60	79.70	38.25	445.66	15.79
28	4.01	35.82	4.49	40.31	17.40	80.95	14.67	317.10	11.78
29	4.02	47.45	13.97	61.42	17.60	80.60	76.20	461.73	6.12
30	4.05	50.24	12.98	63.22	17.87	80.47	83.53	414.43	5.71
31	4.58	23.26	16.47	39.73	16.97	81.27	50.52	366.22	7.18
32	4.72	27.91	4.99	32.90	14.60	83.60	8.98	586.72	14.79
33	4.51	44.04	8.65	52.69	16.87	81.33	45.21	572.43	15.15
34	3.90	37.68	16.47	54.15	18.10	80.20	82.18	409.06	8.93
35	4.09	38.14	14.97	53.12	17.40	80.95	129.03	252.84	2.80
36	4.35	28.84	13.98	42.82	18.00	80.30	42.66	293.01	6.94
37	4.04	43.26	14.97	58.23	18.13	80.10	61.97	383.15	9.34
38	4.45	52.41	13.31	65.72	17.70	80.60	29.41	869.82	16.65
39	4.07	40.01	13.97	53.98	15.80	82.43	34.50	647.34	10.70
40	4.10	28.84	13.97	42.82	18.30	80.05	14.74	799.22	11.46
41	5.42	18.02	0.31	18.33	16.10	82.10	3.37	548.45	22.97
42	4.10	43.61	0.00	43.61	18.13	80.20	19.09	693.25	20.02
43	5.07	23.23	0.46	23.69	17.00	81.15	2.74	767.89	32.28
44	4.96	23.23	0.93	24.16	15.60	82.68	3.29	682.37	23.87
45	5.03	24.18	0.00	24.18	16.73	81.43	2.35	780.38	28.42
46	5.31	21.33	0.00	21.33	15.60	82.50	1.72	489.63	27.19
47	5.19	21.81	0.00	21.81	15.50	82.75	0.70	556.63	27.73
48	4.93	20.86	0.93	21.79	15.07	83.10	1.95	359.79	19.31
49	4.04	33.66	5.11	38.76	19.00	79.40	8.98	530.54	11.04
50	3.49	54.99	4.64	59.63	19.27	79.10	77.39	653.58	10.82
51	4.11	40.77	4.64	45.41	17.00	81.20	50.75	835.88	20.60
52	4.10	25.60	0.93	26.53	19.00	79.30	106.72	339.47	4.63
53	4.18	28.92	1.86	30.78	15.33	82.80	58.75	591.75	10.81
54	4.02	45.11	10.96	56.07	19.05	79.25	24.33	226.65	6.76
55	3.68	28.19	9.46	37.66	17.73	80.53	134.43	299.22	2.85

56	4.80	15.04	3.98	19.02	15.80	82.47	28.51	370.72	13.06
57	4.98	16.92	6.48	23.39	15.73	82.50	51.27	412.89	18.80
58	3.91	10.34	8.97	19.30	16.30	81.93	46.48	126.65	5.08
59	5.36	10.81	5.48	16.29	16.45	81.75	3.74	384.45	23.63
60	5.25	16.92	5.48	22.40	15.87	82.33	1.12	506.02	42.31
61	4.11	28.67	13.95	42.61	19.00	79.40	54.94	400.16	16.37
62	5.02	19.27	6.97	26.24	15.60	82.53	25.37	412.89	19.01
63	4.66	23.97	16.94	40.90	19.00	79.30	9.21	267.77	14.33
64	4.41	19.27	10.46	29.73	19.40	79.00	21.63	495.23	26.72
65	4.32	28.18	6.42	34.60	16.30	81.90	15.84	431.15	20.18
66	3.46	23.49	3.67	27.15	18.10	80.15	94.83	180.92	3.31
67	4.10	28.18	4.12	32.31	17.50	80.85	276.93	204.26	4.13
68	4.12	37.58	2.75	40.33	16.80	81.45	323.53	497.66	6.83
69	4.47	28.18	5.96	34.14	16.60	81.60	13.68	625.46	22.52
70	3.62	67.64	4.12	71.76	18.50	79.70	66.00	675.32	30.22
71	3.59	36.17	5.96	42.13	18.40	79.85	30.05	876.46	18.75
72	4.73	28.17	5.50	33.67	14.60	83.50	7.03	410.03	15.64
73	3.56	38.51	2.75	41.26	19.50	78.80	181.33	620.41	8.35
74	4.48	23.49	0.92	24.40	18.10	80.15	2.91	591.78	32.26
75	4.49	25.36	2.29	27.65	15.80	82.40	96.08	507.35	14.94
76	4.46	25.36	1.83	27.20	16.40	81.85	93.00	509.05	9.55
77	4.27	21.61	4.58	26.19	16.80	81.45	55.90	619.66	13.79
78	4.62	19.73	4.12	23.85	15.80	82.45	5.76	281.23	19.20
79	3.64	52.14	7.79	59.93	19.70	78.60	339.12	787.89	7.88
80	4.79	25.36	5.50	30.86	15.70	82.55	5.75	810.70	26.97
81	4.22	35.23	3.21	38.44	16.47	81.77	12.33	845.36	49.92
82	4.21	32.88	4.58	37.46	15.50	82.65	9.26	326.92	21.17
83	4.08	23.49	3.21	26.69	16.60	81.70	3.51	306.26	19.78
84	3.72	56.36	3.67	60.03	19.60	78.75	77.62	511.09	10.60
85	3.73	34.76	4.12	38.88	17.87	80.33	107.01	288.57	9.21
86	4.07	44.62	7.33	51.96	17.50	80.70	26.48	870.77	30.26

87	4.84	21.14	3.67	24.80	16.80	81.40	12.79	201.14	13.87
88	4.09	54.02	4.12	58.14	18.70	79.55	57.92	495.98	19.08
89	4.83	17.38	5.04	22.42	15.60	82.50	7.33	229.00	20.12
90	3.75	52.61	5.04	57.65	19.40	78.90	23.56	595.59	27.32
91	4.36	33.77	5.03	38.81	15.90	82.35	107.62	308.76	13.48
92	3.54	38.94	3.66	42.60	18.40	79.80	59.72	487.25	10.95
93	4.62	26.74	4.12	30.86	16.60	81.75	5.38	395.62	28.40
94	3.74	34.76	6.87	41.63	18.80	79.55	41.71	689.31	19.20
95	3.47	46.50	5.96	52.45	18.10	80.20	62.55	644.40	28.50
96	3.38	46.03	5.96	51.99	18.60	79.70	31.01	555.46	26.92
97	3.85	50.72	10.54	61.26	19.70	78.65	1.20	199.00	18.26

TSS: Total Soluble Solids; HMF: Hydroxymethylfurfural;

Table S2. Adjustments of the PLS models for the validation dataset of the physicochemical parameters of honey samples from different botanical and geographical origins using mid-infrared spectroscopy data.

Parameters	LV	R	RMSE	RPD	RER
pH	3	0.78	0.28	1.63	6.05
Free acidity	3	0.01	8.39	1.01	3.97
Moisture	3	0.74	0.87	1.50	4.90
Lactonic acidity	2	0.81	3.17	1.75	6.59
TSS	2	0.76	0.82	1.56	5.07
Total acidity	3	0.64	9.16	1.33	4.37
Diastase	2	0.61	6.96	1.28	3.78
Proline	2	0.29	156.14	1.06	4.30
HMF	3	0.01	48.85	0.72	2.26

Fig. S1 Spectral regions selected by *i*PLS to predict pH in honey samples with MIR spectroscopy data.

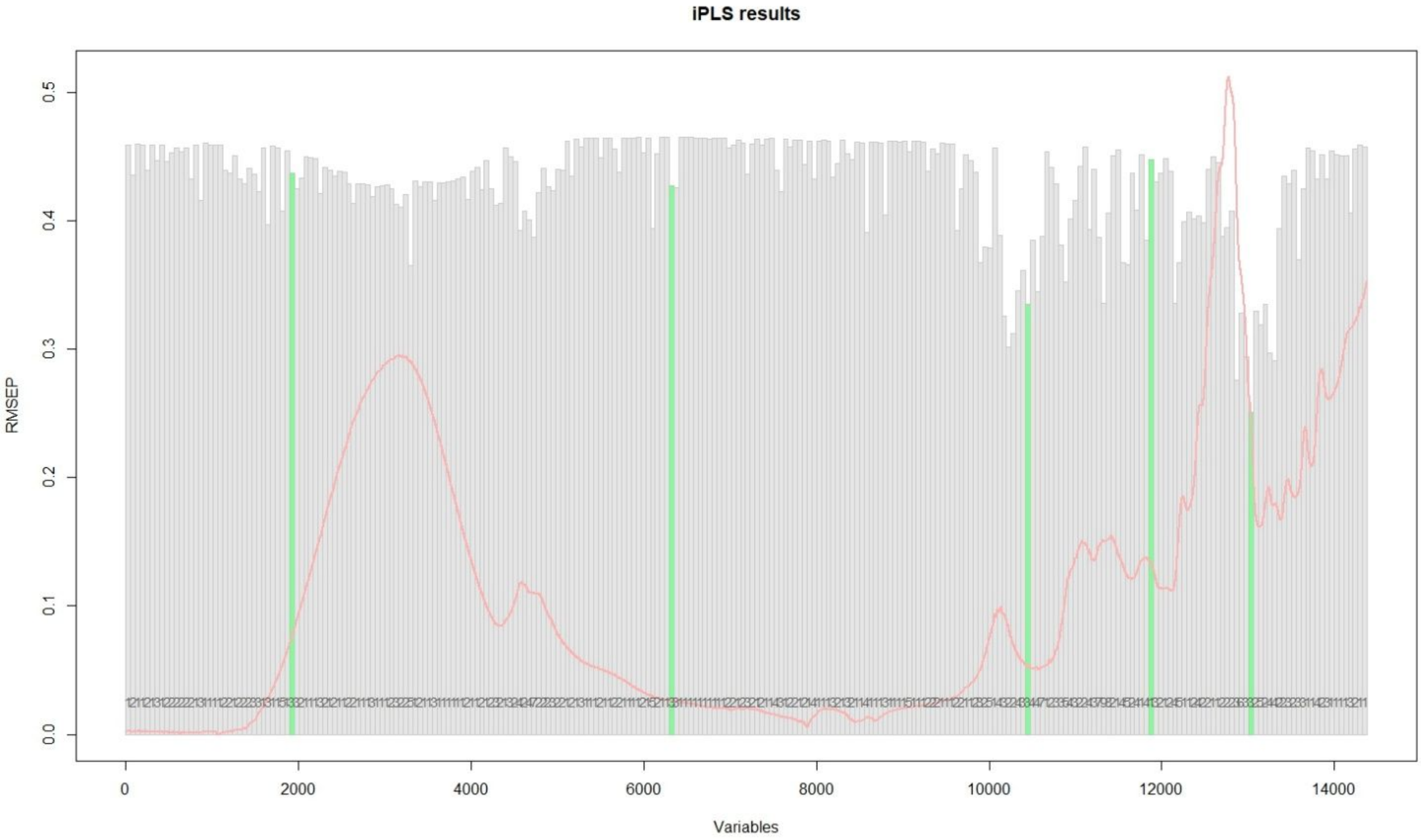

Fig. S2 Spectral regions selected by *i*PLS to predict Free acidity in honey samples with MIR spectroscopy data.

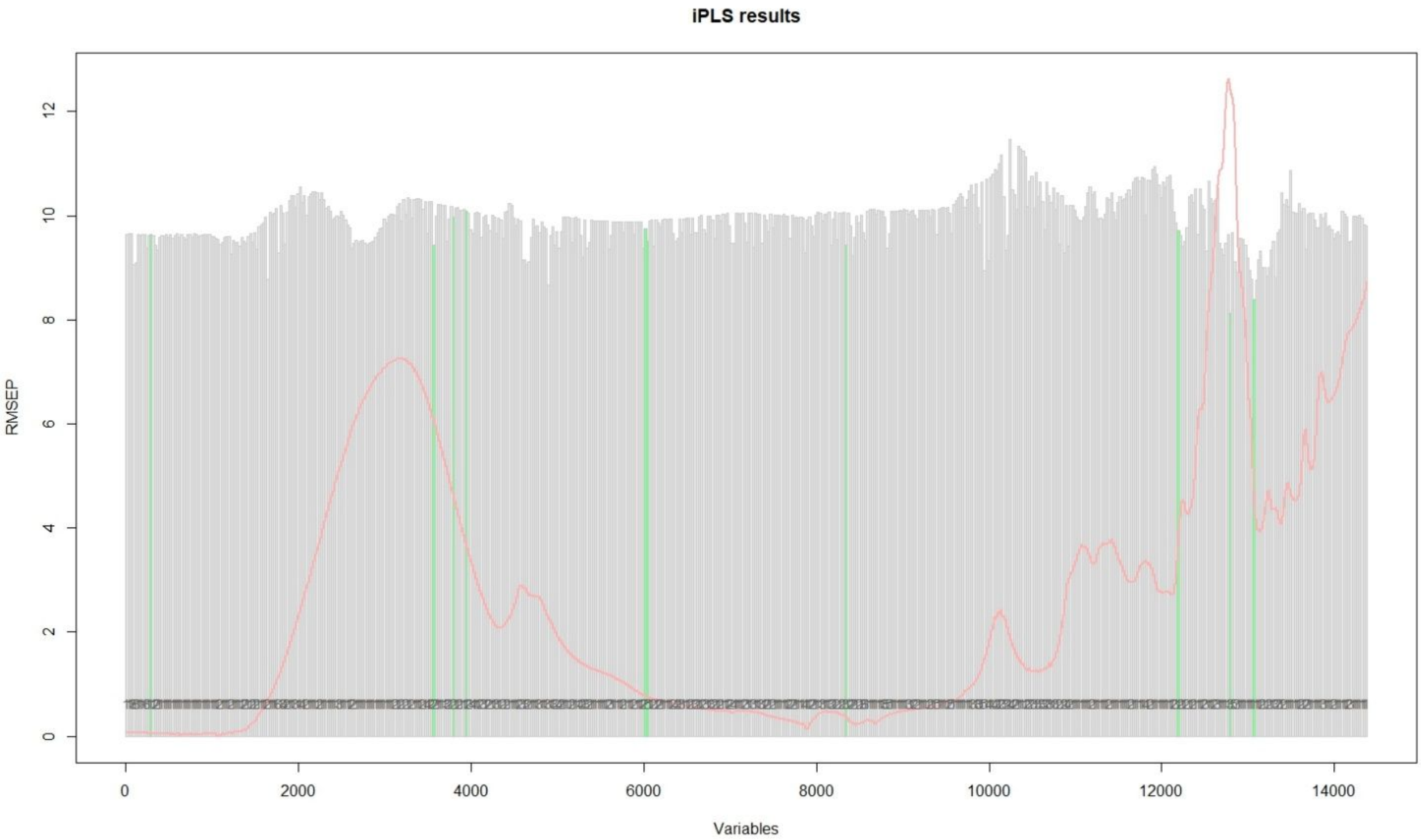

Fig. S3 Spectral regions selected by *i*PLS to predict Moisture in honey samples with MIR spectroscopy data.

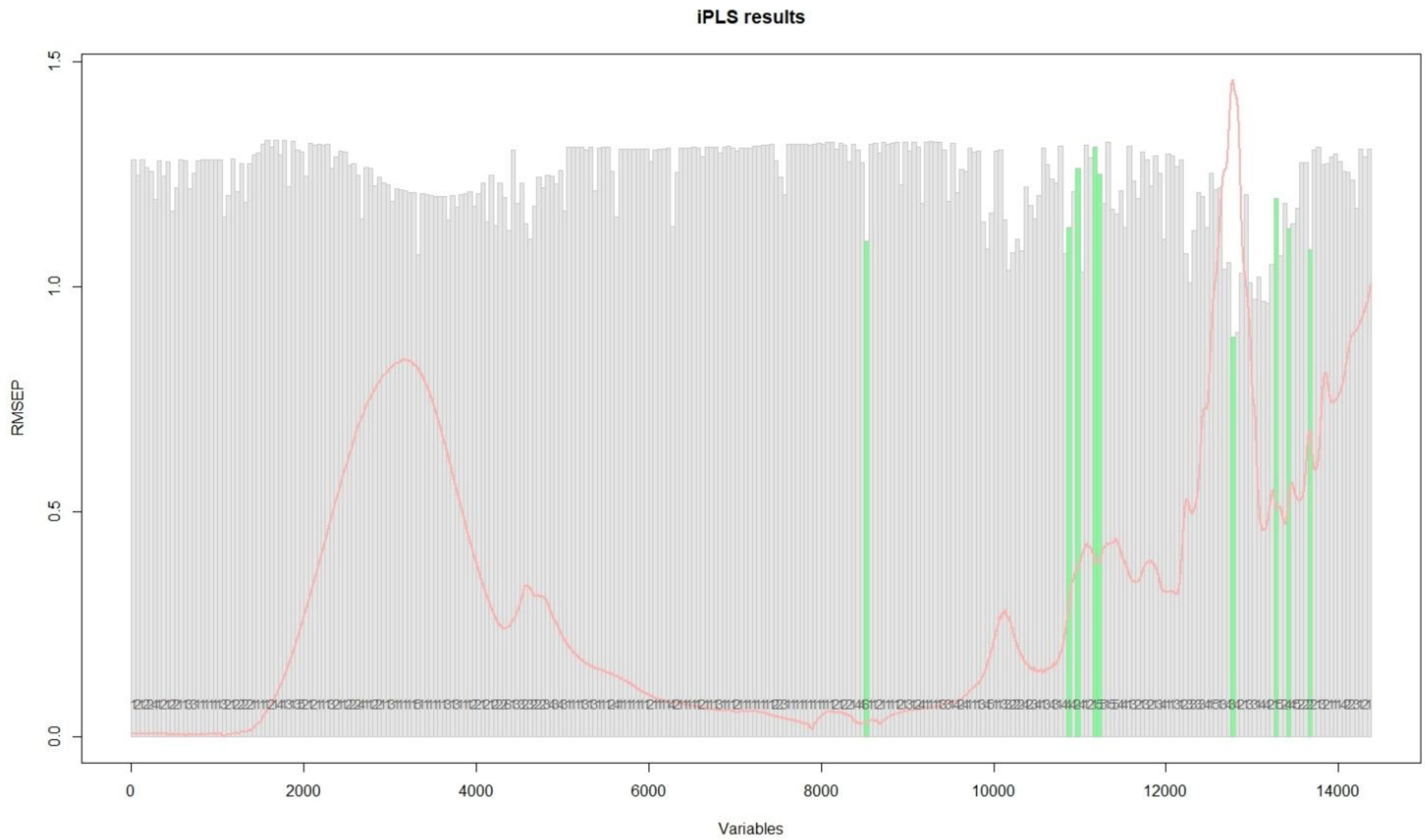

Fig. S4 Spectral regions selected by *i*PLS to predict Lactonic acidity in honey samples with MIR spectroscopy data.

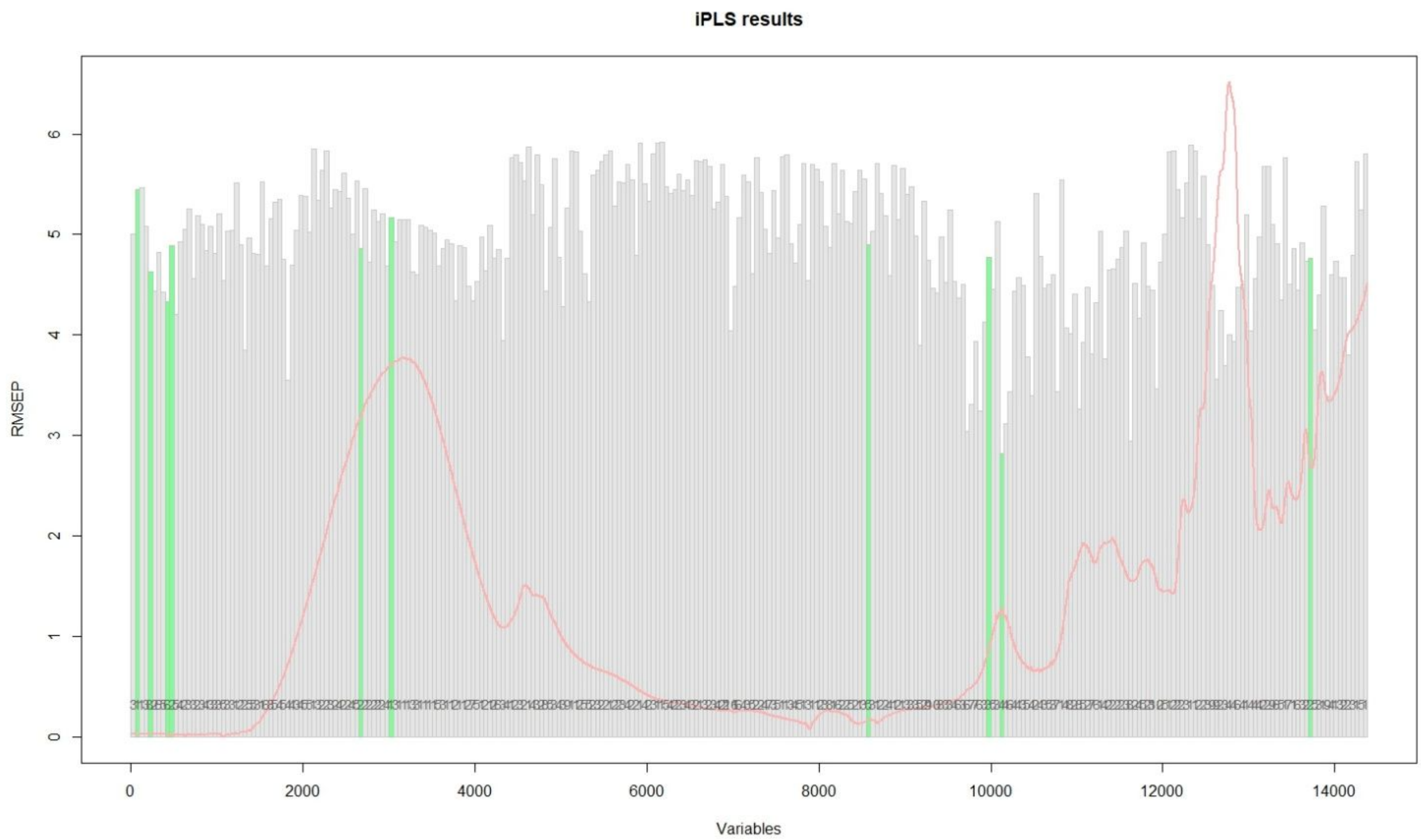

Fig. S5 Spectral regions selected by *i*PLS to predict Total soluble solids in honey samples with MIR spectroscopy data.

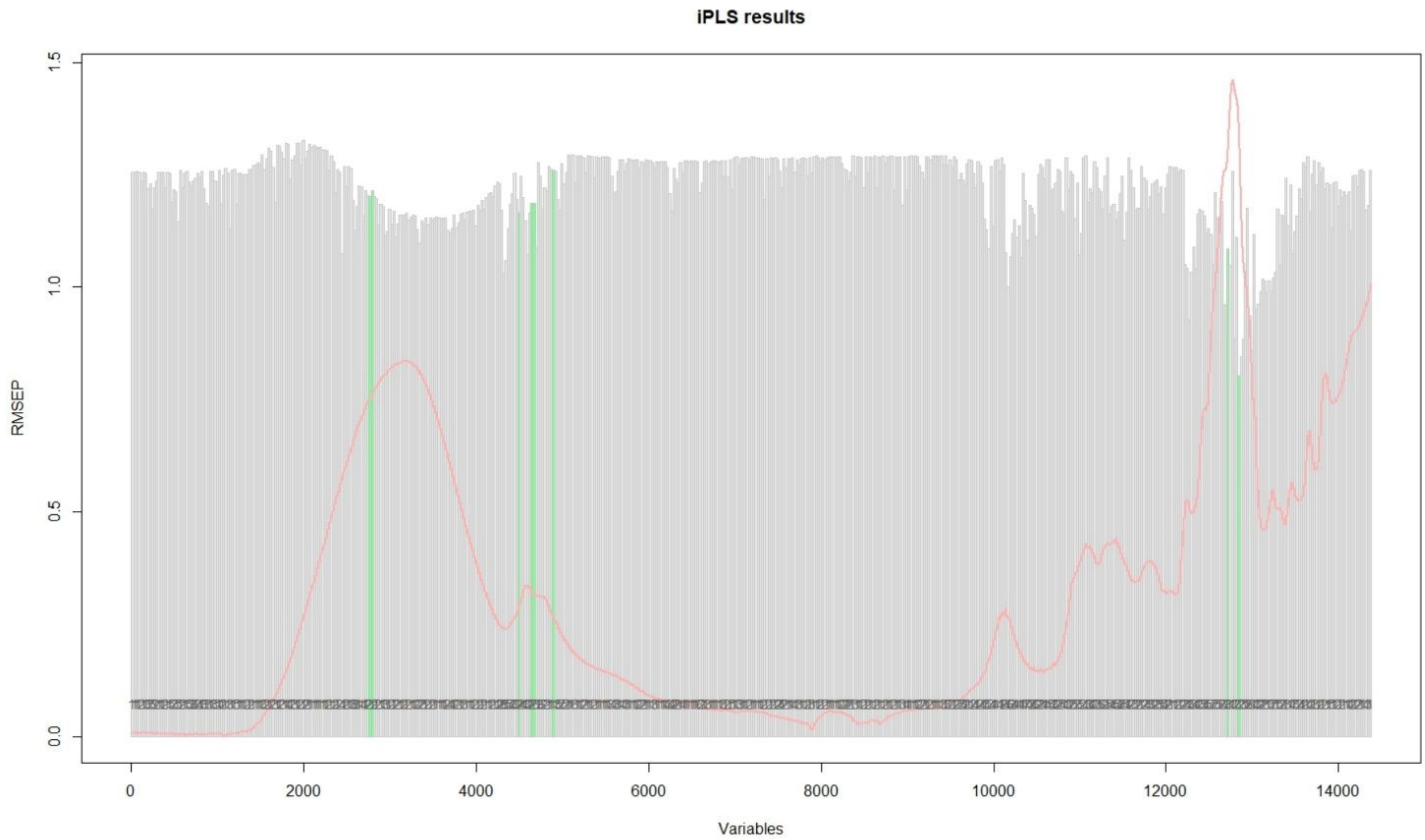

Fig. S6 Spectral regions selected by *i*PLS to predict Total acidity in honey samples with MIR spectroscopy data.

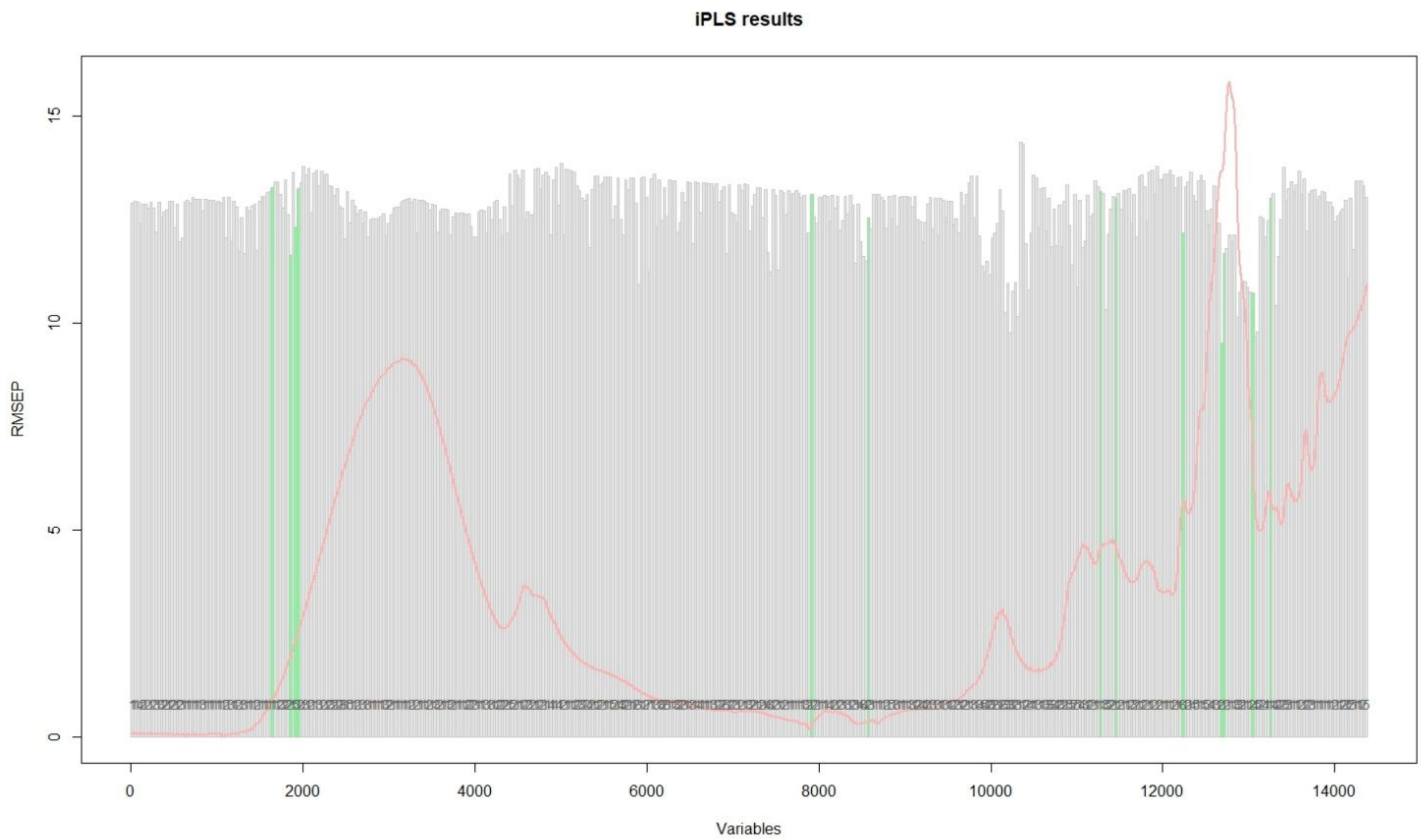

Fig. S7 Spectral regions selected by *i*PLS to predict Diastase in honey samples with MIR spectroscopy data.

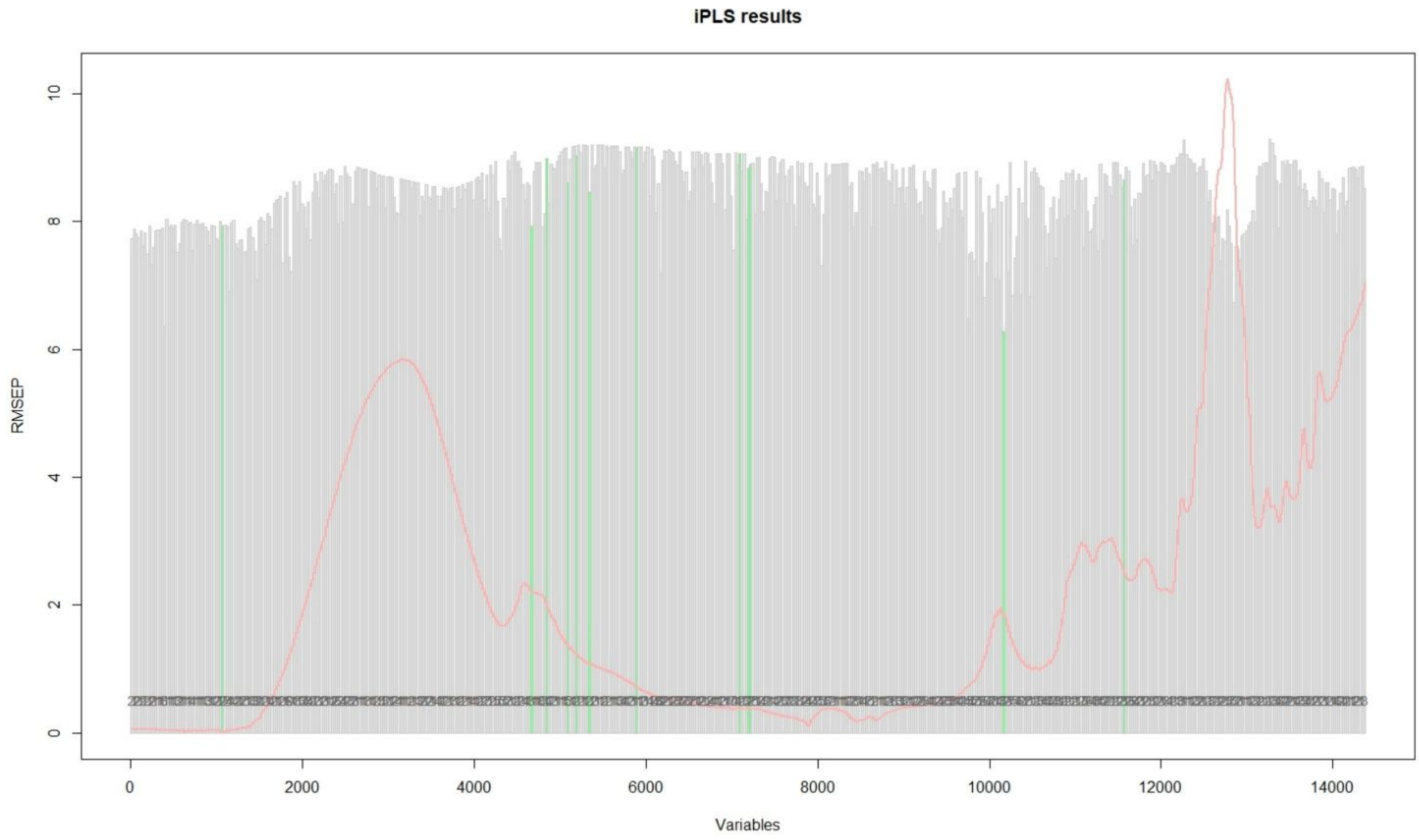

Fig. S8 Spectral regions selected by *i*PLS to predict Proline in honey samples with MIR spectroscopy data.

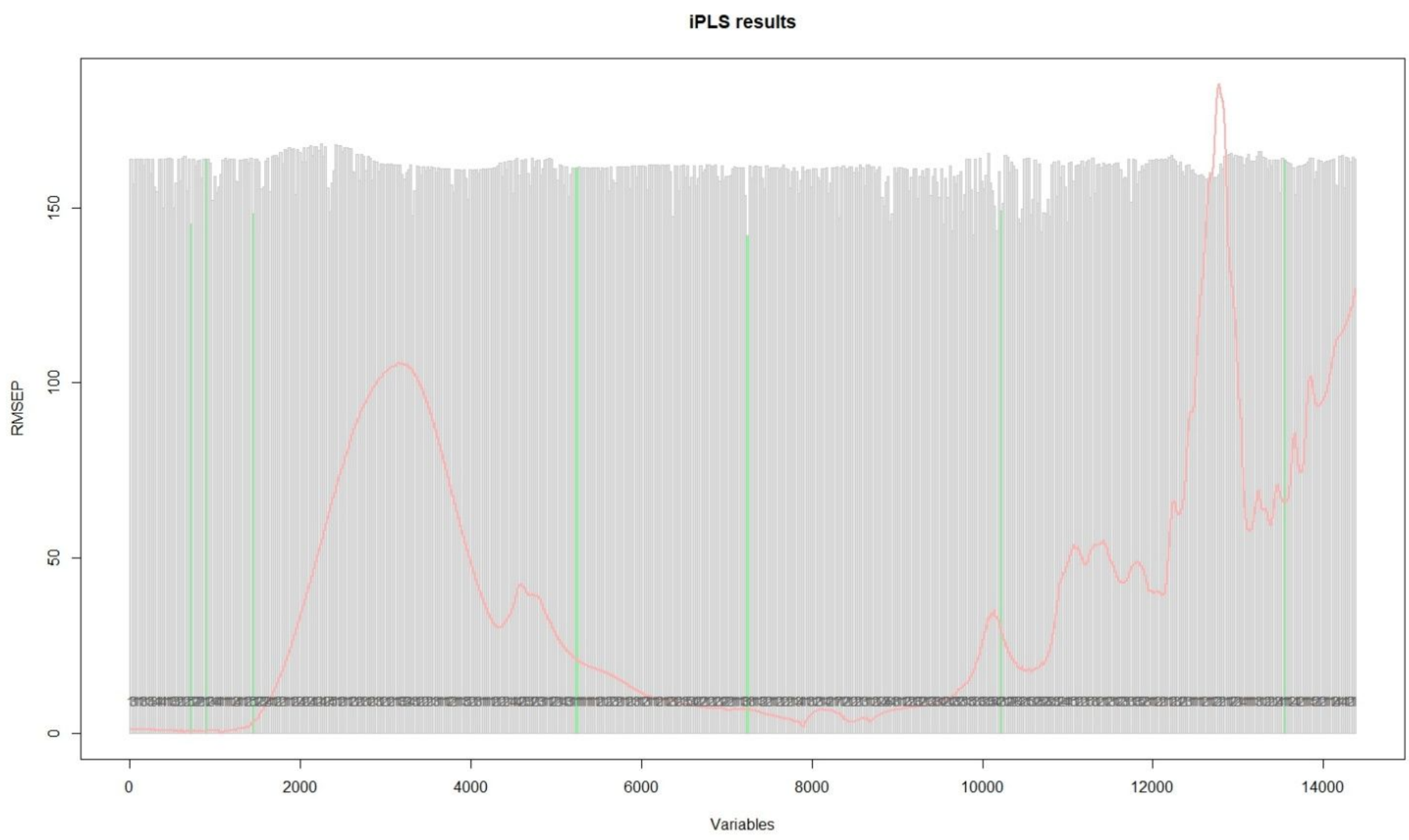

Fig. S9 Spectral regions selected by *i*PLS to predict Proline in honey samples with MIR spectroscopy data.

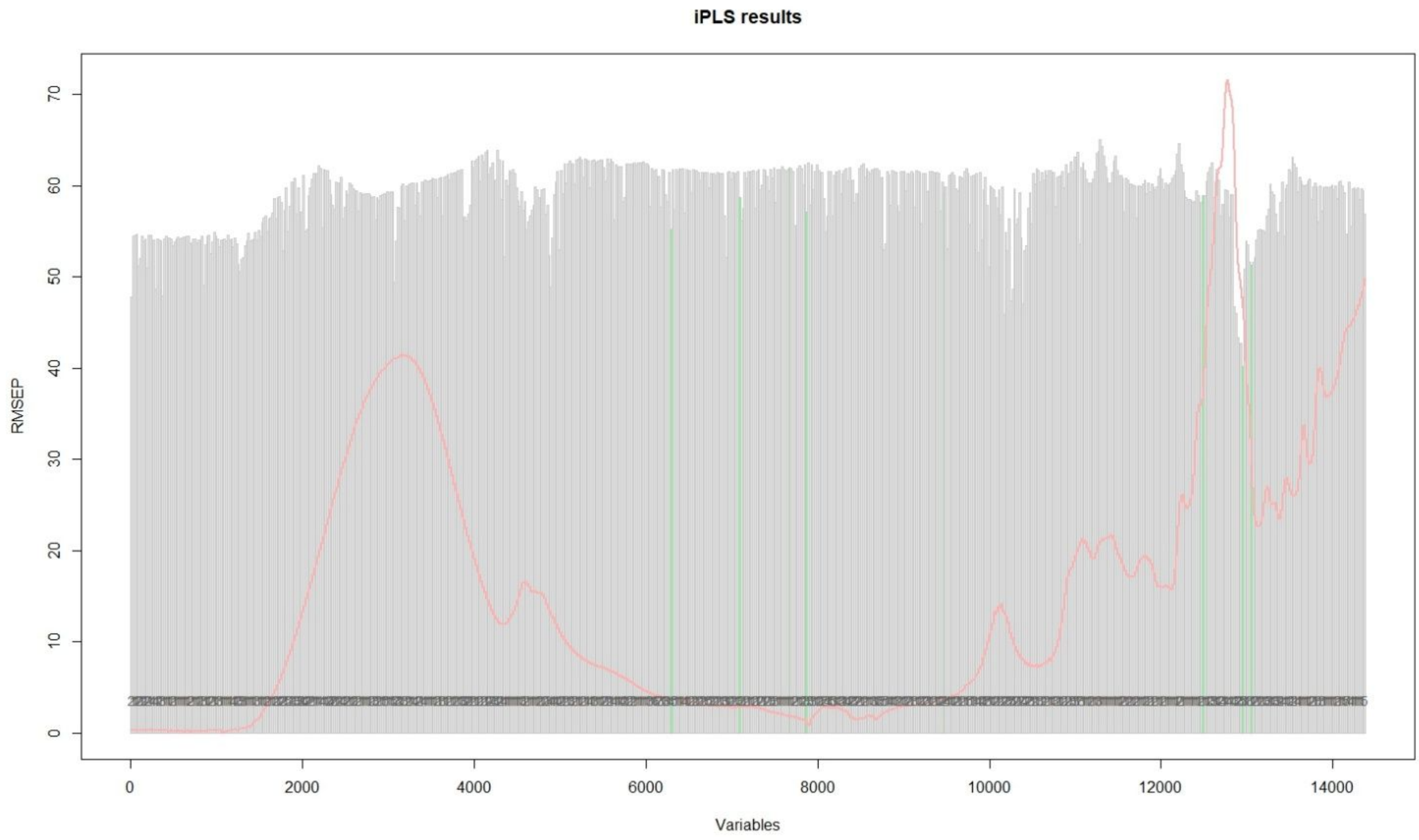

iPLS results
RMSEP
Variables
0
10
20
30
40
50
60
70
0
2000
4000
6000
8000
10000
12000
14000
